# Perioperative hypothermia in pediatric patients operated in a tertiary care center: Incidence and correlates

**DOI:** 10.12669/pjms.36.4.456

**Published:** 2020

**Authors:** Osman Esen, Gulseren Yilmaz, Nevin Aydin

**Affiliations:** 1 Osman Esen, Department of Anesthesiology & Reanimation, University of Health Sciences, Kanuni Sultan Suleyman Hospital, Istanbul, Turkey; 2 Gulseren Yilmaz, Department of Anesthesiology & Reanimation, University of Health Sciences, Kanuni Sultan Suleyman Hospital, Istanbul, Turkey; 3 Nevin Aydin, Department of Anesthesiology & Reanimation, University of Health Sciences, Kanuni Sultan Suleyman Hospital, Istanbul, Turkey

**Keywords:** Hypothermia, Postoperative, Temperature, Thermoregulation

## Abstract

**Background & Objectives:**

Hypothermia, described as temperature < 35°C, is a frequent condition encountered in patients operated under general anesthesia. It is associated with significant morbidity and mortality. We aimed to estimate its incidence and to investigate the conditions associated with hypothermia in pediatric patients.

**Methods:**

This prospective clinical study was carried out in the operating theatre of a tertiary care center between August 2015 and September 2015. A total of 108 pediatric patients who underwent various surgical procedures that lasted for more than 30 minutes were enrolled. Baseline demographic data, types of surgical procedures, duration of operations, preoperative and perioperative body temperatures were recorded. The incidence of hypothermia and its possible correlates were sought.

**Results:**

Our series consisted of 108 children (77 males, 71.3%; 31 females, 28.7%) with an average age of 6.08±5.09 years were included in the study. There was no case diagnosed with hypothermia in the preoperative, perioperative and postoperative periods. Patients in American Society of Anesthesiologists classification (ASA) three group had significantly higher preoperative body temperatures compared to those in ASA-1 and ASA-2 groups (p = 0.027). The postoperative body temperature in patients receiving intravenous fluid replacement was significantly lower (p=0.017).

**Conclusion:**

For pediatric patients scheduled for surgical interventions, we recommend close monitorization and follow-up of body temperature, implementation of preventive measures to avoid hypothermia and routine perioperative heating. Avoidance of hypothermia may prevent hazardous consequences of postoperative hypothermia.

## INTRODUCTION

Hypothermia is a frequent condition that may occur in operating theater as a consequence of general anesthesia and surgery. The body’s core temperature is under the influence of the interaction between heat loss and heat gain. The vulnerability of the patient to a cold environment in the operating room together with the anesthesia-induced deterioration of thermoregulatory control mechanisms may lead to hypothhermia.^1^-^3^ Hypothermia may function as a double-edged sword, since it may be both beneficiary but also harmful in surgical patients. In this context, it may be protective against the deleterious effects of cerebral ischemia and malignant hyperthermia; however, it may also lead to an increase in perioperative wound infection via vasoconstriction and impairment of immunity.^2^-^4^

The adverse effects associated with hypothermia are tremor, coagulopathy, prolonged duration of drug action and diminution of resistance against infections. Fortunately, perioperative warming may alleviate or avoid complications associated with hypothermia. Thus, treatment and prevention of perioperative hypothermia appears to be an important part of perioperative care.^5^-^7^

Hypothermia is detected in almost 70% of patients in the intraoperative period, and it may be affected by several parameters such as anesthetic agents, room temperature, age, systemic diseases and intravenous administration of cold solutions.^8^ Hypothermia is defined as a reduction in body temperature below 35°C, which corresponds to a level of body temperature below the normal range, and the body is incapable of producing essential heat to maintain physiological functions.^6^,^9^,^10^

Surgery usually brings about the risk of vulnerability to a cold environment, administration of unwarmed intravenous fluids, and evaporation from surgical incision site. Nevertheless, since thermoregulatory mechanisms can normally preserve the core temperature within physiological limits, none of these factors alone may cause hypothermias. However, surgical stress, anesthesia, and cool environment in the operation theatre may disrupt the activity of thermoregulatory mechanisms, which maintains body temperature within normal range.^11^ Thus, a better understanding of the impact of anesthesia is required to estimate, prevent, and manage hypothermia and its consequences.

Hypothermia is a common condition detected in patients operated under general anesthesia, and it may constitute a remarkable risk factor for morbidity and mortality. Purpose of this study was to estimate the incidence of hypothermia and to determine the circumstances associated with hypothermia encountered in pediatric patients who underwent surgery in the operating room.

## METHODS

This prospective clinical study was carried out in a tertiary care hospital between August 2015 and September 2015 following principles of Helsinki Declaration was provided. The approval of the local institutional review board (Ref.No.: 2015/2 no and April 30, 2015) and written informed consent of both parents of patients were obtained. These clarifications and signatures took place in the patients’ rooms, on the day of surgery, before administration of pre-anesthetic medication, when required. This study was implemented by the collaboration of anesthesiology & reanimation and pediatric surgery departments in our tertiary care center. Power calculations based on our pilot study with 20 patients revealed that (Preoperative body temperature: 36.6 ± 0.2 mm vs. postoperative 30th minute body temperature: 36.4 ± 0.4, effect size 0.78, alpha error: 0.5 power: 0.95) at least 43 patients were required for an adequate sample size.

Pediatric patients (age ≤ 16 years) who are scheduled for a surgical intervention that is expected to last for more than 30 minutes were enrolled in the study. Patients transferred to the operating room in incubators and children with a predisposition to temperature changes, such as thyroid and neurological disorders, extreme weight, ASA classification IV to VI and axillary body temperature under 36°C or over 37.1°C when entering the operating room were excluded. The recovery time was defined as the time interval between the arrivals of the patients to the recovery room until their Aldrete scores were 10.^12^

The body temperatures of the patient as well as the ambient temperatures were recorded to determine the incidence of hypothermia and to avoid the circumstances associated with hypothermia. The temperatures and humidity of the preparation, operation and recovery rooms were also recorded.

The anesthesiology team who were responsible for the induction of anesthesia were blinded to the study data. The data collected at pre- and postoperative periods were recorded by the same anesthesiologist. Demographic features, and information about the surgical procedure, body and ambient temperatures before, during and after the surgical intervention were recorded. The body temperatures were measured by the nursing staff with the same thermometer (*ThermoScan^®^, infrared tympanic thermometer, Braun, Germany*) applied on the tympanic membrane. Hypothermia was described as the reduction of body temperature below 35°C.^6^,^9^ The type of surgeries, methods used for anesthesia, heating in the perioperative period, the type and amount of intravenous fluid administered, the amount of blood loss and duration of the surgical procedures were recorded. The body temperature at the time of entry to the recovery room was noted. Furthermore, the time interval from entrance to the discharge of the subjects from the recovery room, type of heating if postoperative heating was performed as well as the presence of tremor was noted.

The ambient temperatures in the operating room were recorded by healthcare personnel who were blinded to study data. The types of anesthesia were classified as general anesthesia, neuraxial anesthesia, peripheral nerve block, and general anesthesia together with neuraxial anesthesia. Surgical procedures were categorized as open abdominal, inguinal, closed, endoscopic, head & neck, and other procedures.

### Statistical analysis

Analysis of data was performed using “IBM SPSS Statistics 20” program. The normality of the data distribution was assessed with the Kolmogorov-Smirnov test. Variables with a normal distribution were analyzed using the parametric tests, whereas variables without a normal distribution were evaluated with non-parametric tests. The correlation between selected variables was tested by using the Spearman Correlation analysis. Two independent groups were compared with Mann-Whitney U test, while the Kruskal-Wallis test was employed for comparison of more than two independent groups. Categorical variables were compared with the Fisher Exact test. Quantitative variables were expressed as mean, standard deviation, median and interquartile range. Data were analyzed within a confidence interval of 95%, and the level of significance was set at a p-value less than 0.05.

## RESULTS

A total of 108 children (77 males, 71.3%; 31 females, 28.7 %) with an average age of 6.08 ± 5.09 years were included in the study. The average body-mass index (BMI) was 17.17 ± 6.89 kg/m^2^. The average values for room and body temperatures measured in the preoperative, perioperative and postoperative periods are shown in Table-I There was no case diagnosed with hypothermia in the preoperative, perioperative and postoperative periods.

**Table-I:** Descriptive and clinical information of our series.

Variable	Average (mean ± SD)	Range (min-max)
Age (years)	6.1±5.1	0.1-17.0
Body mass index (kg/m^2^)	17.2±6.9	3.10-35.8
Preoperative room temperature (°C)	22.40±0.86	19.0-24.0
Temperature of heater (°C)	42.90±0.58	38-43
Variable		n (%)
Gender	Male	77 (71.3)
	Female	31 (28.7)
American Society of Anesthesiologists (ASA) classification	1	75 (69.5)
	2	20 (18.5)
	3	13 (12.0)
Perioperative heating	No	6 (5.6)
	Yes	102 (94.4)
Monitorization of temperature	No	97 (89.8)
	Yes	11 (10.2)

SD: Standard deviation.

The investigation for the relationship between demographic variables and preoperative body temperature yielded that there was no difference between males and females (Mann-Whitney U-test, p=0.362). However, patients in ASA-3 group had significantly higher preoperative body temperatures compared to those in ASA-1 and ASA-2 groups (Kruskal-Wallis test, p=0.027). Spearman correlation analysis revealed that there was no association between preoperative body temperature and preoperative room temperature (p=0.647) or BMI (p=0.803).

The duration of operation was positively correlated with the duration of stay in the recovery room (p < 0.001); however, there were no associations between the duration of operation and postoperative body temperature (p = 0.062), and body temperature at the end of operation (p=0.051) (Spearman correlation analysis).

There was a significant correlation between the duration of operation and preoperative body temperature (p=0.029) and intraoperative body temperature on 30^th^ minute (p=0.007). However, the duration of the surgical procedure was not correlated with perioperative body temperature on 1^st^ hour (p=0.759), and 2^nd^ hour (p=0.312).

The duration of surgery was significantly longer in patients with significant blood loss (p < 0.001) and patients who received intravenous fluid replacement such as erythrocyte suspension or fresh frozen plasma (p < 0.001). The postoperative body temperature in patients receiving intravenous fluid replacement was significantly lower (36.8 ± 0.24 vs. 36.3 ± 0.27; p = 0.017). However, intravenous fluid replacement was not correlated with perioperative body temperature on 30^th^ minute (p=0.092) and 1^st^ hour (p=0.733).

Patients who underwent perioperative heating had significantly higher body temperature at the end of the operation (36.7 ± 0.26 vs. 36.5 ± 0.21; p = 0.045). The method of heating (sheets, warm air, radiant heater) did not affect the body temperatures on 30^th^ minute (p=0.099), 1^st^ hour (p=0.553), 2^nd^ hour (p=0.384), and at the end of surgery (p=0.422).

There was no significant difference in perioperative body temperature on 30^th^ minute (p=0.419), 1^st^ hour (p 0.810), 2^nd^ hour (p=0.340), at the end of the operation (p = 0.329) and in the postoperative period (p=0.392) between patients with and without blood loss.

There were no correlations between preoperative operating room temperature and body temperature on 30^th^ minute (p=0.065), 1^st^ hour (p=0.607), 2^nd^ hour (p=0.054) during operation, at the end of the operation (p=0.744), and postoperatively (p=0.873).

## DISCUSSION

Patients who underwent various surgical procedures are prone to many factors that may influence the thermoregulatory mechanisms and lead to postoperative hypothermia. These factors include the temprature of the operating theater, intravenous fluids, blood transfusions, and antiseptic skin solutions as well as the anesthesia. The anesthesia may affect the thermoregulatory responses and inhibit the afferent inputs, thereby decrease the temperature threshold for thermoregulatory responses. Several factors such as surgery lasting for ≥ 2 hours, extremes of age, trauma, abdominal surgery, thoracic surgery, massive transfusions, and massive blood or fluid loss increase the risk of perioperative hypothermia. Inadvertent perioperative hypothermia prolongs the recovery time and also increases blood loss, surgical site infections and hospital stay.^1^

Our results yielded that the incidence of hypothermia for 108 pediatric cases operated during two months in the operating theater of a tertiary care center was 0%. This finding may be attributed to the increased awareness and sensitivity for warming pediatric patients before, during and after the surgery. Perioperative care of the pediatric surgical patients was more intensive and meticulous than adult patients, and this may be a factor leading to the lack of detection of hypothermia in our series.

Unavoidable heat loss is a common condition seen in patients receiving anesthesia. The causes of heat loss involve prolonged surgical procedures, intraoperative significant blood loss, massive fluid replacement and room temperatures less than 23°C.^13^ In the literature, paying special attention and close monitorization are recommended for prevention of hypothermia in elderly.^14^ Children who constitute the extremes of age groups together with the elderly deserve implementation of additional care measures such as routine perioperative heating against hypothermia. In relevant literature, it was reported that hypothermia occurred more frequently after prolonged surgical procedures and operations that involve large body spaces.^15^,^16^

Preoperative evaluation including ASA classification may provide important clues to determine patients who are under risk for hypothermia. Our findings indicate that careful follow-up of fluid balance and refraining from unnecessary intravenous fluids are important points to be taken into account. Perioperative heating is a useful and simple method that may be useful to avoid hypothermia in pediatric patients.

Even though body temperature is accepted as an important component of vital signs, results of a study indicated that only 19.4% of patients receive monitorization of body temperature during surgery.^17^ Monitorization of body temperature can be accomplished using various methods including nasopharyngeal, cutaneous, tympanic and rectal routes.^18^

Perioperative skin warming is supposed to decrease the initial post induction hypothermia, intraoperative hypothermia, and postoperative tremor. Moreover, a single hour of preoperative skin surface warming may diminish the rate at which core hypothermia occurred during the first hour of anesthesia. It has been demonstrated that the no-warming group is under remarkable risk for perioperative hypothermia, which may subsequently lead to significant perioperative morbidity. Perioperative systemic warming, in addition to standard, forced warm air intraoperative warming can reduce the likelihood of blood loss and complications.^16^,^19^

In addition to perioperative warming, preoperative warming can be an important adjunctive measure for the prevention of hypothermia. It has been reported that active or passive warming administered for 30 to 60 minutes during the preoperative period was associated with a significant reduction in perioperative hypothermia.^20^ The lack of the detection of any cases of perioperative hypothermia in our study, where the majority of subjects have received perioperative warming, supports that perioperative warming has been successfully employed in our operative theatre. This sensitivity should be maintained and must be applied to adult patients as well to decrease the perioperative morbidity linked with hypothermia.

The ambient temperature and humidity in the operating theatres can be controlled by automated systems and adjustments can be made concerning the patients’ conditions and preferences of the surgical team. The study of El Gamal et al. have revealed that the operating room temperatures less than 23°C were associated with hypothermia.^21^

There had been several reports on the type or mode of warming, and it has been postulated that active warming such as through heated air blanket, can be more effective than passive warming for the accomplishment of normothermia. However, further trials comparing active and passive warming methods are warranted to tailor an algorithm for the avoidance of hypothermia in pediatric patients.^22^

Results of the present study demonstrate that monitorization of body temperature and implementation of measures for avoidance of hypothermia both in the pre-operative and peri-operative periods may reduce the risk of hypothermia. In the pre-operative period, the presence of risk factors for perioperative hypothermia should be evaluated for each patient and measurements of patients’ temperatures should be carried out at hospital admission. The level of thermal comfort should be determined, and early signs of hypothermia should be sought. In case of the presence of such a finding, documentation and communication with the anesthetic and surgical teams is crucial. In the pre-operative period, the performance of passive measures of thermal care such as maintenance of the room temperature ≥ 24°C and establishing active heating for hypothermic patients without any delay should be considered. It has been reported that pre-heating for a 30 minutes before the surgery may diminish the risk of subsequent intraoperative hypothermia. These simple, practical and effective methods can prevent serious complications and morbidities that result from hypothermia. The temperature of the operating rooms is another critical point for the maintenance of normothermia. The target value for the operating theatre temperature should be close to the higher limits of normothermia. The measures, including passive warming with a sheet and blankets and minimizing the exposure of body surface to the low temperature, should be planned and implemented starting from the pre-operative period. The room temperature should also be controlled closely and the temperature of the operating room must be kept close to the upper limit in order to prevent the development of hypothermia.^23^

On the other hand, there has been contradictory data in the literature suggesting that mild hypothermia per se may not impair respiratory function, nor prolong post-anesthetic recovery in generally infants and children undergoing peripheral surgery.^24^ Since we have not diagnosed any cases with hypothermia, it is impossible to draw further conclusions on the impacts of hypothermia on perioperative physiological functions.

### Limitations of the study

It includes small sample size, the inclusion of only pediatric patients and data restricted to the experience of a single center. Another limitation of this study is that, we aimed to represent the role of preoperative warming on perioperative body temperature in the whole pediatric patient population. We, therefore, enrolled subjects with a wide range of age and BMI. Our findings, therefore, represent data from a wide range of pediatric patient population and cannot be limited to specific subgroups. Further research is required to address the role of preoperative warming on perioperative body temperature in specific subgroups of pediatric patients. In addition, body temperature was measured using a non-contact infrared body temperature technique from the tympanic membrane. Although, body temperature measurements from tympanic membrane have been shown to correlate with the core body temperature, there are also several drawbacks limiting the reliability of this technique. Our findings should therefore be interpreted carefully. The strengths of our study are blinded anesthesiology team for the study and close monitorization of perioperative body temperatures.

## CONCLUSION

There were no pediatric cases diagnosed with hypothermia in the preoperative, perioperative and postoperative periods. We recommend close monitorization and follow-up of body temperature, implementation of preventive measures to avoid hypothermia and routine perioperative heating in pediatric patients scheduled for surgical interventions that may probably last for more than 30 minutes. Avoidance of hypothermia may prevent hazardous consequences of postoperative hypothermia.

### Authors’ Contributions

**OE:** Study design, preparation of manuscript and is also responsible for integrity of research.

**GY:** Data acquisition.

**NA:** Analysis and interpretation of data.
